# Adolescent Self-Reflection Process Through Self-Recording on Multiple Health Metrics: Qualitative Study

**DOI:** 10.2196/62962

**Published:** 2025-04-09

**Authors:** Minseo Cho, Doeun Park, Myounglee Choo, Doug Hyun Han, Jinwoo Kim

**Affiliations:** 1 Department of Cognitive Science HCI Lab Yonsei University Seoul Republic of Korea; 2 HCI Lab Yonsei University Seoul Republic of Korea; 3 College of Medicine Chung-Ang University Seoul Republic of Korea; 4 Business Administration School of Business Yonsei University Seoul Republic of Korea; 5 HAII Corp Seoul Republic of Korea

**Keywords:** self-recording, self-tracking, self-regulation, personal informatics, digital health, qualitative study, grounded theory, adolescents, teenagers, adolescent health, self-reflection, health metrics, behavior change, self-awareness, decision-making, mental health, behavioral health, health management, semi-structured interview

## Abstract

**Background:**

Self-recording is an effective behavior change technology that has long been used in diverse health contexts. Recent technological advancements have broadened its applications. While previous studies have explored its role and benefits in enhancing self-awareness and informed decision-making, relatively little attention has been given to its potential to address the multidimensional nature of health with various health metrics.

**Objective:**

This study investigates the process of self-recording in adolescent health, recognizing the connections between lifestyle behaviors and mental health. Specifically, we aim to incorporate both behavioral and emotional health metrics into the self-recording process. Grounded in self-regulation theory, we explore how adolescents record lifestyle behaviors and emotions, and how they inform and implement health management strategies.

**Methods:**

We conducted a qualitative study using the grounded theory methodology. Data were collected through individual semistructured interviews with 17 adolescents, who recorded their emotions and behaviors over 4 weeks using a prototype application. Analysis followed iterative phases of coding, constant comparison, and theme saturation. This process revealed how adolescents engage in self-recording for behaviors and emotions, as well as their failures and potential system support strategies. We further examined the relevance of the identified themes to theoretical constructs in self-regulation theory.

**Results:**

Under self-regulation theory, we gained insights into how adolescents manage their health through self-recording. The findings suggested variability in the self-recording process, in relation to specific health metrics of lifestyle behaviors and emotions. Adolescents focused on evaluating behaviors for management purposes while exploring the causes underlying emotional experiences. Throughout the health management, which involved modifying behavior or distancing from triggering factors, they monitored progress and outcomes, demonstrating a self-experimental approach. Uncertainty emerged as a barrier throughout the self-regulation process, suggesting that self-recording systems for adolescents should prioritize strategies to address these uncertainties. In addition, the self-recording system demonstrated interventional effects in aiding future planning and fostering a sense of relatedness among users.

**Conclusions:**

This study offers a theoretical framework for adolescents’ self-recording process on diverse health metrics. By integrating self-regulation theory, we suggest a stepwise process from recording lifestyle behaviors and emotions to health management behaviors. Through exploring potential features and health-supportive effects, our findings contribute to the development of digital self-recording systems that address various health metrics in adolescent health.

## Introduction

### Overview

Self-recording has long been practiced in the health domain, and recent technological advancements have expanded its applications [1-3]. Engaging in self-recording and reviewing the recorded data can substantially enhance health by fostering self-awareness and guiding decision-making through reflection [4-7]. Although existing studies often narrow their focus to specific diseases or behaviors, it is essential to view health as a holistic outcome. Lifestyle behavior problems frequently overlap, and mental health closely correlates with these behaviors [8-10]. For the self-recording process to be fully effective, it is crucial to recognize these relationships and integrate diverse health metrics [11-13].

As the importance of considering the multifaceted characteristics of an individual’s health has gained attention, a few studies have explored the visual presentation of various health indicators. In the field of human-computer interaction, researchers have focused on designing effective ways for users to engage with their self-recorded health data [14-17]. For instance, one study involved presenting data in a narrative format, making it easier for individuals to understand and relate to their health information [11]. Another study explored methods to convey and comprehend correlations between different sleep-related health metrics by applying visual cues [18].

These studies are meaningful as they reveal the necessity of a differentiated approach to the various health metrics that constitute health. Furthermore, they suggest practical ways for individuals to engage with and interpret health data. However, the focus of these insights lies on the outcomes, despite self-recording being characterized by concurrent data collection and reflection, thereby necessitating a comprehensive discussion of the process as a whole [19,20]. Specifically, it is crucial to understand how multifaceted health metrics are constructed through self-recording and to explore the unique experiences of success and failure, as well as the strategies used to overcome those challenges [21].

To address this gap, our study aims to observe self-recording patterns for emotional and behavioral aspects of health. We will explore how individuals record these indicators, extract insights, and implement health behaviors accordingly. Furthermore, we will identify factors that facilitate or hinder this process and explore system features that support self-recording for multifaceted health indicators. To ensure our qualitative findings are grounded in evidence-based understandings, we introduce the concept of self-regulation theory [22]. This theory emphasizes self-improvement rooted in self-awareness, providing a robust framework for interpreting our findings.

### Self-Regulation Theory Framework

Self-regulation refers to the ability to manage thoughts, emotions, and behaviors to achieve desired goals, which supports positive health behaviors [23]. Given this study’s focus on self-recordings, we have chosen self-regulation theory developed by Zimmerman for its structured framework of goal setting through reflection and subsequent performances [22]. Zimmerman’s self-regulation theory is a comprehensive framework that explains how individuals control their own learning and behavior through a cyclical model encompassing three phases: forethought, performance, and self-reflection.

In the forethought phase, learners engage in task analysis by setting specific goals and planning strategic approaches to achieve them. This phase is characterized by self-motivation beliefs, including self-efficacy, expected outcomes, intrinsic interest, and goal orientation. During the performance phase, learners use self-control strategies such as self-instruction, imagery, attention focusing, and time management to maintain concentration and motivation. Simultaneously, they practice self-observation by systematically monitoring their performance and recording their progress to ensure alignment with their goals [22,24,25].

The self-reflection phase involves self-judgment, where learners critically evaluate their performance by comparing it to their predefined goals and standards and engage in causal attribution to discern the reasons behind their successes or failures [22,26]. Following this, self-reaction occurs, where learners experience emotional responses such as satisfaction or dissatisfaction and make adaptive inferences to refine their strategies and goals. Positive outcomes lead to enhanced self-efficacy and further motivation, while negative outcomes prompt re-evaluation and adjustment of strategies to better align with future goals.

This continuous feedback loop ensures that each phase informs and improves the others, enabling a dynamic process of learning and self-improvement. By organizing our study around the phases of self-regulation and their associated constructs, we anticipate investigating the dynamics of adolescents’ perceptions and behaviors during self-recording. We aim to analyze and gain deeper insights into specific processes leading to the benefits of self-recording, aligning with a series of phases outlined in the theory.

### Self-Recording Intervention for Adolescent Health

Self-recording provides health benefits by increasing awareness of emotions and behaviors, which is often used as an important therapeutic step across various clinical domains [27-29]. As smartphones become more integrated into daily life, many adolescents already use mobile apps to monitor and improve their health [30-32]. Leveraging these technologies, studies on digital self-recording systems have shown that adolescents readily engage with technology and exhibit high compliance [33-35].

Adolescence is an important developmental stage characterized by cognitive, physical, psychosocial, and emotional growth [36]. This stage serves as a transition from childhood to adulthood, yet it is also a fragile period often involving exposure to stressful life events that can lead to decreased well-being and impaired mental health [37]. With the significance of early intervention in addressing mental health challenges, particularly as adolescents develop coping mechanisms for daily stressors and negative emotions, studies have demonstrated the efficacy of digital self-recording in emotional well-being [38-42].

In a case study, a self-recording system increased positive mood and coping strategies in the adult population under stress [43]. Participants reported enhanced emotional self-awareness and were able to internalize therapeutic strategies. Similarly, a randomized controlled trial examined the relationship between self-recording and depressive symptoms in adolescents [44]. Through self-recording, adolescents learned to identify and differentiate emotions within different contexts, effectively communicate their emotions to others, and make informed decisions [44,45].

Evidence also suggests that digital self-recording can benefit physical health [23]. Adolescents’ behavioral patterns can significantly impact their health and increase the risk of developing chronic diseases in adulthood [10,46]. Addressing these behaviors is crucial, as they often co-occur and emerge during adolescence [8,47-49]. A study on overweight or obese adolescents revealed clinically significant weight loss achieved by self-recording dietary actions [50]. A meta-review study highlighted that behavior change techniques involving self-recording can decrease sedentary behavior and increase moderate to vigorous physical activity [51,52].

As outlined above, the use of digital self-recording systems has shown promise in enhancing emotional self-awareness, coping strategies, and overall health behaviors [53]. Digital systems offer key advantages over traditional instruments, such as enhancing motivation and adherence while reducing participant burden [54,55]. However, empirical evidence presents a different perspective, revealing that digital systems are prone to disengagement, with significant variability in time-dependent self-reporting observed both across studies and among individuals [56].

Therefore, rather than relying on assumptions, it is important to thoroughly examine user behavior to identify the barriers and facilitators influencing engagement. This study aims to observe adolescents’ behavior while using a digital self-recording system and to analyze the entire process as they record their emotions and daily activities over a 4-week period.

## Methods

### Participants

A total of 17 adolescents, aged 12-18 years, participated in this study, comprising 3 males and 14 females. The eligibility criteria required participants to be middle or high school students in Korea with no difficulties using mobile phones, as our digital self-recording system operated on mobile technology. These criteria were selected to reflect the influence of school life and peer relationships on adolescents’ emotions and daily activities [57,58].

In addition, we aimed to ensure that our study’s observations aligned with the experiences of typical Korean adolescents.

### Study Design

Participants were instructed to record their emotions and daily activities over 4 weeks using an application prototype. The application was developed based on a pilot study that examined how adolescents recorded their emotions and daily activities over a 2-week period, as well as their expectations for a digital self-recording system [59]. The application served as a data platform, designed to collect both passive and active digital phenotypes. Passive digital phenotypes were collected through device sensors where the application was installed, while active digital phenotypes were gathered from the voluntary records provided by participants. After using the application, the participants took part in a semistructured interview lasting approximately 60 minutes to share their experiences with the application.

Emotion recording uses the Ecological Momentary Assessment method, which captures real-time emotional experiences [51,60]. Participants received notifications prompting them to complete a questionnaire up to 8 times a day. These prompts included inquiries about mood disorders such as depression, anxiety, and stress, along with an evaluation of overall mood. Notifications were scheduled at key events throughout the day: upon waking, before and after school, during lunchtime, before and after tutoring, before bedtime, and if the phone was used for more than 30 minutes past midnight. Participants were restricted to recording their emotions only in response to these notifications.

Depression, anxiety, and stress levels were rated on a 5-point Likert scale, where higher scores indicated more severe emotional distress (Multimedia Appendix 1). The format of the question was direct, asking, “On a scale of 1 to 5, how would you rate your current level of depression?” Participants also assessed their overall mood by selecting from a list of mood-related adjectives, such as “happy, sad, or angry.”

**Figure 1:**
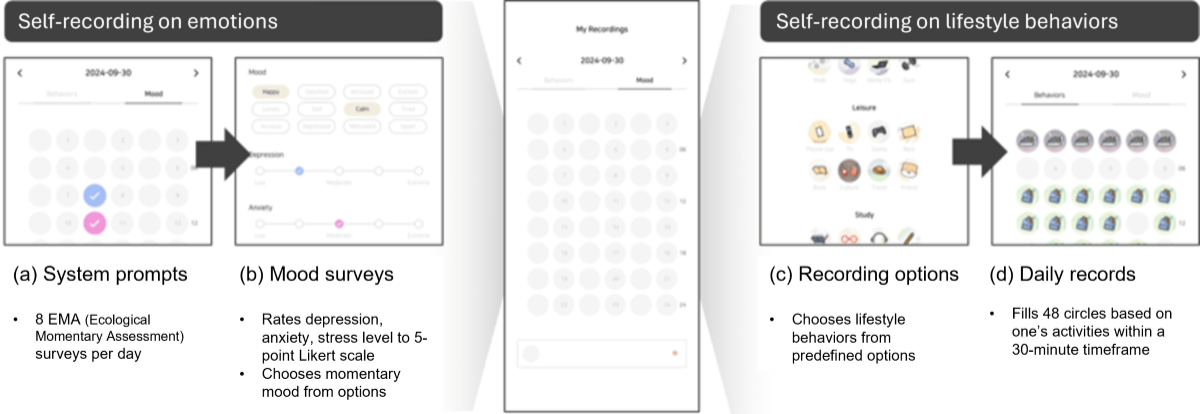
Process of self-recording on the application prototype.

Simultaneously, participants logged their daily activities at 30-minute intervals using the same application (Figure 1). To ensure consistency in data collection and analysis, we offered predefined options that were verified to adequately reflect various aspects of adolescent life. The application featured a daily recording screen with 48 circles, each representing a 30-minute time slot. Upon clicking a circle, adolescents were presented with predefined options categorized under the major headings of sleep, exercise, study, and leisure, with subcategories such as “nap,” “smartphone use,” and “self-study.”

### Data Collection and Analysis

Data collection and analysis followed a systematic, iterative, and concurrent approach, as suggested by Charmaz [61]. Individual semistructured interviews were conducted via video conferencing or telephone, and recordings were transcribed immediately for analysis. This iterative process allowed us to refine interview questions continuously based on adolescents’ responses, ensuring alignment with our research questions. The analysis began with open coding, where segments of the transcripts were coded into initial categories until these categories accounted for all variations in the data. Next, selective coding was applied to identify relationships between categories. Finally, axial coding helped to identify core categories and organize them into broader themes.

After capturing important themes related to our research, we mapped them to the theoretical taxonomy of self-regulation theory. Each theme was compared against theoretical constructs to assess its relevance and validity. To capture the nuanced cognitive processes of adolescents, we used manual coding that allowed for a deeper and more context-sensitive interpretation of the data. The coding process involved multiple researchers working collaboratively through iterative rounds of review to enhance consistency. Each researcher independently read and coded the transcripts line by line, after which they collaborated to resolve discrepancies and reach a consensus. The process was repeated until data saturation was reached, with no new themes emerging from the analysis, and no further interview questions deemed necessary.

### Ethical Considerations

Researchers explained the purpose and procedure of the study and obtained written consent from those who agreed to participate. The study was approved by the Yonsei University institutional review board (IRB number 7001988-202406-HR-2045-05) and all participants received compensation of 100,000 KRW (US $68.14) upon completion.

## Results

### Process of Self-Recording on Emotions and Behaviors

**Figure 2:**
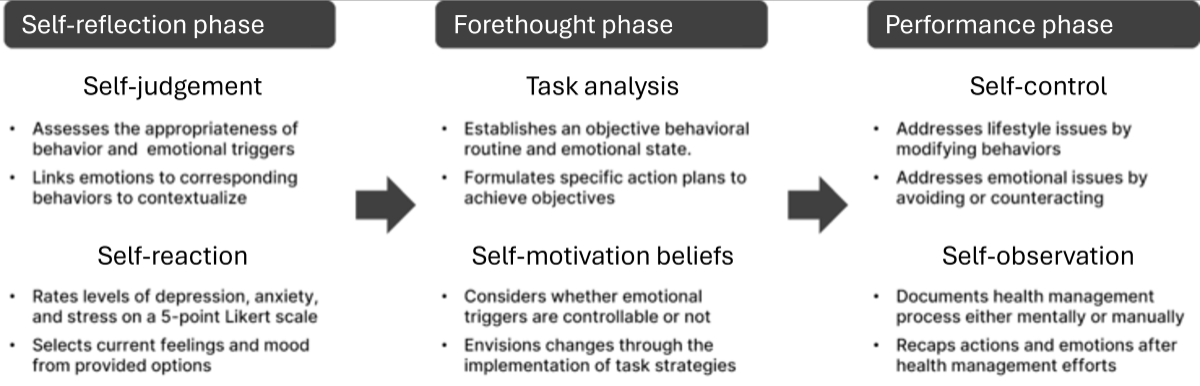
Summary of the self-recording process on emotions and behaviors.

Our study aimed to develop an understanding of the self-recording process that arose while interacting with multidimensional health data. Results revealed stepwise behaviors stemming from self-recording, with significant differences observed between lifestyle behaviors and emotions (Figure 2). Based on the 3 phases and constructs of the self-regulation theory, the research results are summarized as follows.

#### Self-Reflection Phase

The self-reflection phase consisted of two key components: perception and evaluation. Perception entailed acknowledging factual data, while evaluation involved transforming it into meaningful information. Adolescents gained perception regarding emotions and lifestyle behaviors through either self-recording or reviewing records. Subsequently, they evaluated these perceived aspects based on personal criteria.

I recorded how I spent time idling or doing unproductive things, and seeing it visually made me realize how much time I was wasting. It had a bit of a reflective effect, making me reconsider my habits.

P7

It felt like a diary, letting me look back and think, “What was I doing at this time?” I didn’t realize it before, but through recording, I found out that I study in very short, interrupted sessions.

P18

Regarding their behaviors, adolescents mainly perceived the type of behavior they engaged in, its frequency, duration, and the specific time stamps on which they performed the daily activities. By synthesizing these data points, they identified recurring lifestyle behavior patterns and evaluated whether these behaviors aligned with their ideals or universally recommended standards.

In terms of emotions, adolescents primarily perceived the emotional valence and intensity by reviewing their records. Unlike lifestyle behaviors, which focused on appropriateness, the evaluation of emotions is centered on understanding the underlying causes. Adolescents placed greater importance on unraveling the reasons behind their psychological struggles rather than determining their acceptability.

Before using this app, I didn’t often bring my emotions to the surface to think about them directly. But while using the app, when I received an EMA prompt and realized that I felt “irritated,” I would think, “Why am I irritated?” and it helped me find some answers.

P1

During the process of identifying these causes, adolescents identified the causality between life and emotion by integrating information acquired through a series of perception and evaluation processes. If the information obtained from emotional records was insufficient to pinpoint the cause, adolescents turned to additional sources, such as their recorded lifestyle behaviors.

Between 7 and 9 p.m. is exactly when evening self-study starts, and if I tried hard during that time but things didn’t go well, I’d feel anxious because there’s no time left afterward, and I’ve already let so much time slip by. I realized feeling anxious between 7 and 9 p.m.

P17

They would reflect on their behaviors and circumstances surrounding the emotional episodes to gain a deeper understanding of what may have contributed to their emotional distress. In cases where these sources did not provide clarity, adolescents sought external information, such as recent events or experiences, to help contextualize their emotional experiences and identify potential triggers.

#### Forethought Phase

In the forethought phase, adolescents established goals and behavioral strategies to address emotional and behavioral problems identified from previous self-reflection. They noted that emotional disorders not only cause discomfort but also adversely impact behaviors, thereby necessitating resolution. Hence, their overarching goal was to alleviate high levels of depression, anxiety, and stress, with subgoals and specific behavioral strategies varying based on the factors influencing these mood disorders.

When adolescents identified life behavior issues such as irregular sleep, excessive media use, or insufficient leisure as triggers for emotional disorders, they took proactive steps to address these behaviors. Here, the criteria used to evaluate behaviors during the self-reflection phase served as the ideal point to be reached, guiding adolescents’ goal-setting process. Subsequent strategic planning focused on reinforcing positive lifestyle behaviors while modifying or eliminating negative ones.

This study coincided with the exam period, and I noticed that my mood worsened with smartphone use. It made me realize that I experience more stress when I’m not studying much rather than when I’m studying a lot, so I tried to limit my smartphone use.

P1

I noticed that when I’m stressed, I tend to meet with friends more often. It made me feel that spending time with people and taking breaks is really important.

P2

Emotional disorders sometimes originate from factors beyond life behavior, such as external circumstances. In such instances, where adolescents had limited control over the cause, their goal shifted to alleviating the influence of these factors on their emotions. Specific strategies included distancing themselves from influential individuals or situations, engaging in activities aimed at positively altering their emotions, and creating environments conducive to better emotional experiences.

I try to avoid facing it. Given the current situation, I can see that doing more and putting more effort will just lead to more stress, so avoidance seems like the only option.

P16

When I engage in exercises I enjoy, those negative emotions tend to disappear. For example, when I play sports or listen to my favorite songs, I feel like those emotions gradually lessen.

P27

#### Performance Phase

In the performance phase, adolescents pursued their previously established goals and plans. When emotional disorders were linked to lifestyle behaviors, their strategies focused on changing them. Conversely, if emotional disorders were triggered by external factors, such as interpersonal conflicts or stressful situations, adolescents aimed to mitigate the impact by distancing themselves from the causes or counteracting negative emotions with positive ones.

In both scenarios, adolescents recalled and implemented their goals and strategies. Some managed this mentally, continuously reflecting on their objectives and progress. Others used more structured approaches, documenting their plans and tracking their progress with tools like planners and notes. This documentation provided a tangible reminder of their commitments and a clear framework for their actions.

I started to use a note-taking app on my phone. I write down the parts that I think are problematic and consider how to address them. When I have free time, I try to make progress on those issues as much as possible.

P2

In the morning, I often feel very tired which makes it hard to stay motivated. There are many times when I end up wasting that time or not being productive. However, by becoming more aware of those feelings, I kept reminding myself to take proper breaks. This helped me study more effectively with a clearer mind.

P11

This implementation was followed by the cycle of self-monitoring and adjustment, which enabled adolescents to stay focused and adapt their strategies as needed. Specifically, when modifying lifestyle behaviors, adolescents assessed whether their actions led to actual improvements. In cases where they directly addressed the emotional states, they evaluated whether their strategies successfully induced positive emotions or reduced negative ones.

If their actions did not produce the expected effects, adolescents iteratively adjusted their plans to identify effective actions to achieve desired outcomes. This trial-and-error process, also termed self-experimentation, involves testing, reinforcing, or revising plans based on their outcomes. This approach allowed adolescents to continually refine their strategies, optimizing their management of both lifestyle behaviors and emotional well-being (Table 1).

I tried going to exercise in the morning and in the evening to find out which time would allow me to be more consistent.

P17

**Table 1:** Findings on self-recording process and relevant theoretical constructs.

Self-regulation theory				Interview findings
**Phase and** **Constructs**				
	**Self-reflection**			
		Self-evaluation	Lifestyle behaviors were assessed for appropriateness Emotions were examined for underlying triggers	
		Causal attribution	Behavioral and emotional data were integrated to contextualize emotions	
		Self-reaction	Outcomes for health management efforts were evaluated post performance	
	**Forethought**			
		Goal setting	Lifestyle behaviors were managed through behavior modification Controllable causes were addressed through behavior execution Uncontrollable causes were mitigated to reduce their impact	
		Strategic planning	Positive behaviors were reinforced, and negative behaviors were adjusted Positive emotions were amplified, and negative emotions were neutralized	
		Self-efficacy	Emotional triggers were assessed for controllability	
	**Performance**			
		Task strategies	Actions leading to positive emotions were consistently pursued Steps to avoid triggers for negative emotions were deliberately taken	
		Self-recording	Progress was recorded either mentally or manually	
		Metacognitive monitoring	Goals and strategies were revised based on improvements	

### Identified Barriers in Self-Recording Process

Despite the findings aligning with previous studies and theories, not all participants successfully engaged in the process of self-regulation. While some adolescents effectively reflected on their emotions and behaviors through self-recording, set and pursued health management goals, and reflected on the outcomes, others partially or entirely failed in the process. The factors contributing to these failures were analyzed as follows, with the overarching theme of “uncertainty.”

#### Uncertainties in Self-Reflection Phase

During the phase of self-reflection, where adolescents perceive and evaluate their emotions and lifestyle behaviors, distinct uncertainties emerged. Regarding lifestyle behaviors, uncertainty arose from unclear evaluation criteria. Adolescents often struggle to assess the appropriateness of their behavior when faced with ambiguous standards. Without objective criteria, such as socioculturally accepted norms or clear personal goals, the assessment became challenging, significantly impeding their transition to the next phase of self-regulation.

Another uncertainty was about the causes of emotions. Adolescents often fail to accurately identify or misidentify the causes of their emotions. Compounded with the psychological instability of adolescence, pinpointing the reasons behind emotional triggers was particularly difficult, hindering effective emotional issue resolution. For example, an adolescent stressed from lack of sleep might incorrectly focus on securing enough sleep while overlooking the underlying issue of late-night smartphone use, thereby failing to resolve the emotional problem.

I don't really understand why I'm feeling these emotions, so I can't pinpoint the cause.

P28

#### Uncertainties in Forethought Phase

The forethought phase presented challenges regarding the uncertainty in the task setting. Adolescents struggled to determine which specific aspects of their behavior or circumstances needed alteration to improve their emotional well-being. This uncertainty about what to change led to indecisiveness and a lack of direction in their goal-setting and planning efforts. Furthermore, they frequently lacked the necessary resources—emotional, cognitive, or environmental—to devise potential changes in their lifestyle behaviors, leading some to abandon planning altogether.

I used to have hobbies that I enjoyed, but I find it difficult to make time for them. The easiest thing to do is just use my smartphone. I know that the smartphone isn't the most effective solution, but I don’t really know of any other alternatives.

P7

If I had information about what kind of lifestyle I should have at my age and how I should behave, I think it would be helpful for managing my emotions and daily life.

P8

There was also uncertainty about outcome expectations. The unpredictability of outcomes often made adolescents hesitant to set goals for managing their emotional problems. They noted that if their efforts did not lead to improvements, it would confirm their insecurities. Faced with this uncertainty, they often relied on instant, verified means of emotional management, such as gaming, phone use, or sleeping. Although they expressed a desire to engage in other activities, they feared that exploring new methods would take too long, ultimately wasting their time and effort.

I find it hard to know if I can control and manage my emotions through my actions, so sometimes I just move on.

P14

The effectiveness of management techniques varies from person to person. Even if a certain approach works for some, it might not work for others, which makes me hesitant to follow advice.

P2

Even when I know I should exercise, I find it hard to feel the urgency since health issues tend to arise later. So, when I hear advice to exercise, I tend to think, “I'll do it later,” and end up ignoring it.

P1

#### Uncertainties in Performance Phase

In the performance phase, there was uncertainty in the process. Adolescents sought external acknowledgment and validation to reassure themselves of following the correct steps in managing their emotions. This external validation served as positive reinforcement, encouraging persistence even when immediate results were not apparent. Without this reinforcement, many adolescents lost motivation. Some reported to have experienced a chain reaction of demotivation occurring if one person stops updating progress.

I believe the best way to feel validated is through recognition from others. It seems that I can affirm myself through the actions of others who acknowledge me.

21

Furthermore, we could observe uncertainty about the effectiveness. Adolescents required tangible evidence that their efforts were yielding expected changes. Doubts about abilities and strategy arose when significant improvements were not observed, making it difficult to sustain participation in problem-solving strategies. While self-tracking provided some benefits, if the tracking results mainly highlighted failures, their self-efficacy diminished, leading them to give up on making further efforts altogether (Table 2).

If recording gives me a sense of achievement in reaching my goals, I think it would reduce the annoyance and make me want to continue managing things.

P11

I got to think that if studying might make me feel bad, I should bring some chocolate to cheer myself up. Being aware of when my emotions fluctuate has really helped me in that regard.

P8

**Table 2:** Findings on self-recording barriers, explanations, and relevant theoretical constructs.

Barriers				Explanation		Constructs
**Phase** **and uncertainty**						
	**Self-reflection**					
		Uncertainty in evaluation	Lacks a personal sense of direction Lacks external criteria for evaluation		Self-evaluation	
		Uncertainty about causes	Struggles to identify emotional triggers Misidentifies emotional triggers		Causal attribution	
	**Forethought**					
		Uncertainty in strategies	Unsure about necessary adjustments Struggles to devise practical changes in routine		Goal setting and strategic planning	
		Uncertainty about expected outcomes	Finds it challenging to anticipate outcomes Reluctant to experiment with new approaches		Self-efficacy and outcome expectations	
	**Performance**					
		Uncertainty in progress	Finds it difficult to recognize ongoing behaviors		Self-Instruction Imagery	
		Uncertainty in improvements	Struggles to be aware of own emotions Repeated failures reduce motivation		Metacognitive monitoring	

### Design Implications for Self-Recording System

Based on the observations of the study participants’ self-recording processes, we identified design implications for system features aimed at reducing uncertainties and thereby preventing failures. During the analysis, participants were divided into 2 groups according to their successes and failures in self-regulation. For adolescents who successfully self-regulated, we focused on inferring the cognitive processes and strategies they used to succeed. Conversely, for those who failed, we investigated the reasons behind their failures and their expectations regarding the system.

#### Design Implication 1: Introducing Unaware Self

To reduce uncertainty in self-reflection, it was essential to help adolescents identify the causes of their emotional problems. This understanding was a crucial precursor to developing and implementing strategies for addressing these issues. Our interview findings revealed that adolescents who successfully identified their emotional triggers recorded their levels of depression, anxiety, and stress while simultaneously contemplating the reasons behind them. This immediate reflection on the cause at the moment of recording prevented delays that might cloud their understanding.

From these observations, we derived a design implication: developing a feature that enables adolescents to record their lifestyle behaviors alongside their emotions. For instance, incorporating prompts like “Why did you experience this emotion?” can provide space for adolescents to note the causes of their feelings. Even if they do not respond to the prompt every time, the presence of the question can nurture the habit of self-reflection by consistently encouraging them to consider the reasons behind their emotions.

In addition, some adolescents expressed interest in a system that could predict emotional triggers preemptively. Recent research suggests that digital phenotypes—behavioral and psychological data collected through digital devices—can be used to develop artificial intelligence models capable of predicting emotional problems. By developing a function that leverages both active digital phenotypes (data recorded directly by adolescents) and passive digital phenotypes (data automatically collected through device sensors), we can create a system that significantly aids in identifying the causes of adolescents’ emotional triggers.

#### Design Implication 2: Managing Controllable Factors

Next, the uncertainty regarding the forethought phase required tailored strategies. According to our interview results, the causes of emotional problems often stemmed from factors that individuals could control, such as personal behavior, as well as factors beyond their control, such as the influences of others or external circumstances.

Adolescents responded to controllable factors by modifying their behavior, emphasizing the importance of developing feasible action plans tailored to their daily lives. Those who successfully planned did not stop at the abstract intention of “modifying behavior,” but instead considered specific actions they could incorporate into daily routines. The applicability of these actions was crucial, as it determined the details of how the actions could be carried out.

In a similar context, some adolescents expressed a desire for a system that could collaboratively set goals and behavioral plans with them. In addition, they hoped the system would compare their behavior during emotional problems with their behavior when they were not experiencing such issues, identifying specific areas for improvement. This comparison could highlight patterns and differences in their actions, providing insight into which behaviors might contribute to or alleviate their emotional problems.

Accordingly, a proposed system function could not only predict and present potential emotional triggers but also guide adolescents in modifying their behavior. This could involve showing expected changes in a “What if X” format, where “X” represents a proposed change in behavior. By simulating the expected outcomes of these changes, the system can inform adolescents about actionable behaviors they can adopt in their daily lives. In addition, seeing the potential effects of these changes can help adolescents reconsider their motivation and commitment to implementing these behaviors.

#### Design Implication 3: Managing Uncontrollable Factors

Adolescents often responded to uncontrollable factors by either distancing themselves from the situation, suppressing their emotions, or engaging in activities believed to induce positive emotions. For instance, they may avoid negative emotions by avoiding individuals who caused emotional distress or seek refuge in sleep. However, strategies that involved ignoring situations or emotions were found to be less effective because they failed to address the underlying issues.

Commonly mentioned activities to induce positive emotions among adolescents included smartphone use and interactions with friends. However, socializing often demanded significant time commitments, which was not always feasible. Furthermore, some adolescents perceived that smartphone use exacerbated their emotional problems rather than alleviating them.

Adolescents who effectively managed negative emotions often possessed insight into specific behaviors that reliably elicited positive emotions for them. These individuals had a clear understanding of what activities bring them joy and contentment, as well as the duration required for such actions. As a result, they could respond appropriately to negative emotions by actively seeking out these positive experiences.

Therefore, the possible system function should aim to identify which behavior or context elicits positive emotions for each individual and present them as options accordingly. By unique emotional responses and contexts, personalized recommendations can be generated to address emotional distress. This approach involves suggesting activities known to induce positive emotions, thereby empowering adolescents to proactively manage their emotional well-being.

#### Design Implication 4: Sustained and Dynamic Support

During the performance stage, adolescents faced uncertainties in both the process and its effectiveness. Successful adolescents highlighted the importance of consistently implementing coping plans and engaging in repetitive actions. These practices not only cultivated problem-solving habits but also instilled a sense of control and stability in their lives. Keeping track of their performance through records served as tangible evidence of their commitment, thereby reducing uncertainty in the process. To address these challenges, we recommend incorporating a system feature to monitor plan implementation, enabling adolescents to recognize their progress in addressing emotional problems more effectively.

Furthermore, adolescents sought validation regarding the effectiveness of their coping plans. Reflecting on their emotional experiences enabled them to gain deeper insights into how their efforts influenced their emotional well-being. The system can facilitate this process by prompting adolescents to record and review their emotional responses immediately after taking certain actions. In addition, it can visually demonstrate the effectiveness of the plans by presenting changes in emotions and behavioral data. This feedback mechanism is expected to provide valuable insights and encouragement, motivating adolescents to persist in managing their emotional well-being effectively.

Another important factor in terms of persistence was the way the system interacted. Adolescents recognized that they could succeed or fail in implementing the plan, and sometimes adjustments to the plan were necessary. However, they noted that a system providing a consistent and static response to these varied situations might impede regular performance. Repeated failure to adhere to the plan led some adolescents to adopt a passive attitude toward solving emotional problems, and in severe cases, they even considered giving up.

Therefore, it is essential for the system to provide support, ensuring that adolescents do not feel burdened by their inability to follow the plan. Rather than focusing on what they failed to do, adolescents preferred to be encouraged to try again with reassurance that “it’s okay.” When unable to implement the plan, adolescents were already aware of their shortcomings and sought comfort instead of repetitive confirmation. They also desired assistance in counteracting frustration and learning to recognize small positive changes that may have gone unnoticed (Table 3).

**Table 3:** Self-recording system features to reduce uncertainties.

Identified themes				System features
**Uncertainty type and design implications**				
	**Uncertainty in evaluation**			
		Present data in an intuitive format to facilitate evaluation.	Visualize the current state and goal state simultaneously	
		Support self-evaluation of appropriateness	Provide a variety of information that can serve as assessment criteria	
	**Uncertainty about causes**			
		Support the identification of emotional triggers	Predict potential causes for emotional states based on personal data history	
		Assist in linking behaviors and emotions	Offer contextual data alongside emotional data	
	**Uncertainty in strategies**			
		Assist in determining areas for improvement	Predict and explain behavioral or emotional issues Compare data from periods of good and poor health	
		Outline specific action plans to be executed	Provide suggestions for substituting behaviors within current routine	
	**Uncertainty about expected outcomes**			
		Help understand changes in behavior and emotion	Predict potential changes based on tracked actions and previous data	
	**Uncertainty in progress**			
		Foster metacognition through self-recording	Prompt the recording of behaviors and emotions after specific actions	
		Assist in following self-instructions on progress	Provide external validation of day-to-day efforts	
	**Uncertainty in improvements**			
		Help grasp improvements from effort	Outline changes in behavior and emotion through visualized data	
		Prevent dwelling on failure and suggest success	Responsively highlight positive changes in response to success	

## Discussion

### Principal Findings

This study explored the behavioral and emotional aspects of adolescent health, focusing on how adolescents engage in self-recording as part of their health management. Specifically, using self-regulation theory, we examined the steps adolescents take to establish and implement health management strategies through self-recording, providing a deeper understanding of their perceptions and actions throughout the process.

The findings revealed that the self-recording process varied based on the type of health metric being tracked. Adolescents primarily focused on evaluating the appropriateness of their behaviors and identifying areas for improvement. When it came to emotional aspects, they tended to explore the underlying causes of their emotions. Notably, adolescents often linked their emotional reflections with their lifestyle behaviors, indicating an interconnected relationship between the two.

The strategies adolescents used for managing their health also differed based on the focus of their self-recording. For lifestyle behaviors, they aimed at implementing behavioral changes. For emotional aspects, they first assessed whether they could control the underlying causes of their emotions before developing coping strategies. Factors within their control were addressed through behavior modification, while factors beyond their control were mitigated by minimizing their impact.

Finally, the performance phase of self-regulation could be understood as a process of self-experimentation. Adolescents tracked the outcomes of their health management strategies and adjusted if the results of their management efforts were unsatisfactory. This process highlighted the blurred boundary between the performance and reflection stages, as adolescents continuously adapted their strategies based on ongoing self-assessment.

### Reducing Uncertainty to Enhance Self-Regulation

We also uncovered factors contributing to failures in the self-regulation process, unified under the overarching concept of uncertainty. Throughout the various phases of self-regulation, uncertainty served as a barrier, either complicating or entirely preventing adolescents from progressing to the next phase. The specifics of these uncertainties varied based on the unique characteristics of each self-regulation phase and its associated components.

To address these challenges, we explored system features designed to alleviate uncertainties and support adolescents in engaging effectively with health management through self-recording. The proposed system features aimed to address adolescents’ informational gaps—directly tackling the primary theme of uncertainty. Providing objective criteria for assessing behaviors and offering in-depth interpretations of self-recorded data, especially during emotionally challenging moments, were critical to overcoming these barriers.

Participants suggested incorporating advanced technologies like artificial intelligence to enhance system functionality. They envisioned features capable of predicting emotional triggers and predicting the outcomes of specific actions [62]. Design implications highlighted the need for dynamic recommendations tailored to adolescents’ performance, offering context-rich information to maintain self-reflection, prevent setbacks, and foster resilience. By addressing these needs, the system could promote sustained health management and positive outcomes.

Engaging adolescents in self-recording is essential for ensuring high-quality data and achieving positive health outcomes. To enhance engagement, we recommend reducing uncertainties associated with self-recording [17]. Meeting adolescents’ specific informational needs at each phase of self-regulation is crucial, as these needs are not always immediately clear. We propose that observational studies are essential for gaining deeper insights into adolescents’ behaviors and preferences, enabling the design of systems that effectively address these challenges.

### Self-Recording System and Health Benefits

Our research team identified the interventional effects of the self-recording system, which was the primary tool used in this study. Since no additional intervention materials were incorporated, such as sessions or informational content, the potential effects on physical and psychological health improvements were derived from the interaction between the system and the participants. Adolescents’ feedback revealed that self-recording helped them establish structured planning. As they recorded their lifestyle behaviors, many participants mentally mapped out future actions, with some preemptively writing down their plans and later assessing their actions against their initial intentions.

This was largely due to the system’s interface, which visualized participants’ recordings in a timetable format. The timetable format proved particularly beneficial for adolescents, enabling them to track their daily activities (time use) and evaluate whether these activities were satisfactory (appropriateness of time use). It also provided a clearer understanding of the context surrounding their emotional distress by presenting relatable scenarios. Based on these findings, we propose that digital interventions for adolescents should prioritize visualization features that align with both the targeted health issue and users’ interests [63].

Furthermore, our self-recording tool featured interactive notifications, such as friendly greetings like “Good morning,” “Did you have a good day at school?” and “How was your dinner?” These interactions were generated based on rules we programmed into the system, reflecting the everyday lives of adolescents. Participants reported that these interactions made them feel as though the system was aware of their lives, fostering a sense of relatedness. This feeling of being cared for encouraged greater engagement with the system and provided emotional support, which in turn contributed to their overall health management.

In conclusion, we propose that designing a self-recording tool presents a unique opportunity to promote health management through features such as notifications or prompts [64,65]. In our study, the sense of being cared for and the enhanced self-awareness of one’s health status were shown to have intrinsic health benefits. More broadly, these findings align with the principles of Self-Determination Theory, which emphasizes that meeting basic psychological needs—autonomy, competence, and relatedness—fosters intrinsic motivation. Consequently, we envision integrating health-related theories into the design process to encourage both intrinsic motivations to use the tool and effective health management.

### Limitations and Future Directions

First, the self-recording tool we developed did not include synchronization or integration features with commercially available services, restricting participants to the tool’s preset functions. Participants familiar with other self-recording tools may have the required time to adapt to our system. However, using the same tool for all participants ensured consistency and minimized external influences. In addition, given the lack of existing systems that comprehensively track both emotions and lifestyle behaviors, a customized tool was necessary to address our specific research objectives. Yet, we suggest that future studies could consider starting with tools adolescents already use or exploring the development of systems that incorporate features deemed useful in market products.

Second, the study’s sample size was limited to 17 participants from Korea. This relatively small sample may affect the generalizability and statistical robustness of our findings, potentially introducing bias. However, in grounded theory research, smaller sample sizes are common, with data collection ceasing when no new insights emerge. This aligns with our sample size of 17 participants but remains a limitation for broader applicability. Third, data analysis relied on manual coding, performed by multiple researchers through an iterative process to enhance objectivity. Despite these efforts, unrecognized subjectivity may have influenced the results, particularly in determining saturated themes and aligning findings with theoretical taxonomies. While this approach is standard in qualitative research, further efforts to mitigate subjectivity could strengthen future analyses.

Finally, our study focused on adolescents attending regular classes in Korea, limiting the generalizability of findings due to the sociocultural specificity of this population and the higher proportion of female participants. Adolescents’ emotions and daily behaviors are heavily shaped by their social contexts, including peers and school environments. Consequently, applying the same self-recording tool in different cultural settings may yield varying results. Although the gender imbalance of the participants is a limitation, it also reflects the global trend of higher reported emotional difficulties among female adolescents [66,67]. Yet, we recommend that future research should aim to include more diverse cultural backgrounds and more representative samples to enhance the applicability of findings.

### Conclusions

This study empirically presents a theoretical framework for adolescents’ engagement in the self-recording process of multiple health metrics. By aligning our observations with the constructs of self-regulation theory, we systematically analyze the steps involved in self-recording leading to health management behaviors, focusing on behavior and emotional regulation. We extend our findings to explore the causes of failure and identify design implications for digital self-recording systems to effectively support this process. The study thus informs the development of a digital self-recording system that addresses multiple health metrics for adolescents.

## Appendix

Multimedia Appendix 1 Daily Mood Questionnaire.

## Data Availability

The datasets generated or analyzed during this study are not publicly available due to participant confidentiality and ethical restrictions as they contain sensitive or identifiable information but they are available from the corresponding author on reasonable request.
