# Supplementary material for: Adolescent Self-Reflection Process Through Self-Recording on Multiple Health Metrics: Qualitative Study
Source: J Med Internet Res. 2025 Apr 9;27:e62962. doi: 10.2196/62962 (PMC12018858; doi:10.2196/62962)
Supplement: Multimedia Appendix 1 [file jmir_v27i1e62962_app1.docx]

**Q1.** On a scale of 1 to 5, how would you rate your current level of depression?
*(1 = Very low, 5 = Very high)*

**Q2.** On a scale of 1 to 5, how would you rate your current level of anxiety?
*(1 = Very low, 5 = Very high)*

**Q3.** On a scale of 1 to 5, how would you rate your current level of stress?
*(1 = Very low, 5 = Very high)*

**Q4.** Select all the adjectives that describe how you are feeling right now:

- Happy
- Satisfied
- Annoyed
- Excited
- Lonely
- Sad
- Relaxed
- Tired
- Anxious
- Depressed
- Enthusiastic
- Angry
